# Platelets, diabetes and myocardial ischemia/reperfusion injury

**DOI:** 10.1186/s12933-017-0550-6

**Published:** 2017-05-31

**Authors:** Isabella Russo, Claudia Penna, Tiziana Musso, Jasmin Popara, Giuseppe Alloatti, Franco Cavalot, Pasquale Pagliaro

**Affiliations:** 1 Department of Clinical and Biological Sciences, University of Turin, 10043 Orbassano, TO Italy; 2 Department of Public Health and Pediatric Sciences, University of Turin, Turin, Italy; 3 Department of Life Sciences and Systems Biology, University of Turin, Turin, Italy; 4 Internal Medicine and Metabolic Disease Unit, San Luigi Gonzaga University Hospital, Orbassano, Turin Italy

**Keywords:** Antiplatelet therapy, Aspirin, Cardioprotection, Type 2 diabetes

## Abstract

Mechanisms underlying the pathogenesis of ischemia/reperfusion injury are particularly complex, multifactorial and highly interconnected. A complex and entangled interaction is also emerging between platelet function, antiplatelet drugs, coronary diseases and ischemia/reperfusion injury, especially in diabetic conditions. Here we briefly summarize features of antiplatelet therapy in type 2 diabetes (T2DM). We also treat the influence of T2DM on ischemia/reperfusion injury and how anti-platelet therapies affect post-ischemic myocardial damage through pleiotropic properties not related to their anti-aggregating effects. miRNA-based signature associated with T2DM and its cardiovascular disease complications are also briefly considered. Influence of anti-platelet therapies and different effects of healthy and diabetic platelets on ischemia/reperfusion injury need to be further clarified in order to enhance patient benefits from antiplatelet therapy and revascularization. Here we provide insight on the difficulty to reduce the cardiovascular risk in diabetic patients and report novel information on the cardioprotective role of widely used anti-aggregant drugs.

## Background

Using population-wide preventive strategies, most cardiovascular diseases (CVDs) can be avoided by modifying behavioural risk factors, diet, physical activity and harmful use of tobacco, drugs and alcohol. However, at the individual level, secondary prevention of CVDs in those with established risk factors and treatment of comorbidities, including diabetes, with appropriate medications are necessary. Type 2 diabetes mellitus (T2DM) is an expanding global pandemia characterized by a very high cardiovascular risk [1, 2] and diabetic patients without previous cardiovascular events show similar risk for cardiac mortality as non-diabetic subjects after myocardial infarction [3]. The excess risk for CVDs in patients with diabetes needs a multifactorial prevention, including an effective antiplatelet therapy for the correction of the thrombotic risk [4, 5].

In this mini-review we briefly analyse the issues of antiplatelet therapy, and how the pleiotropic effects of this therapy and platelets *per se* may affect ischemia/reperfusion injury (IRI) in T2DM heart.

## Aspirin function on platelets

**Fig. 1:**
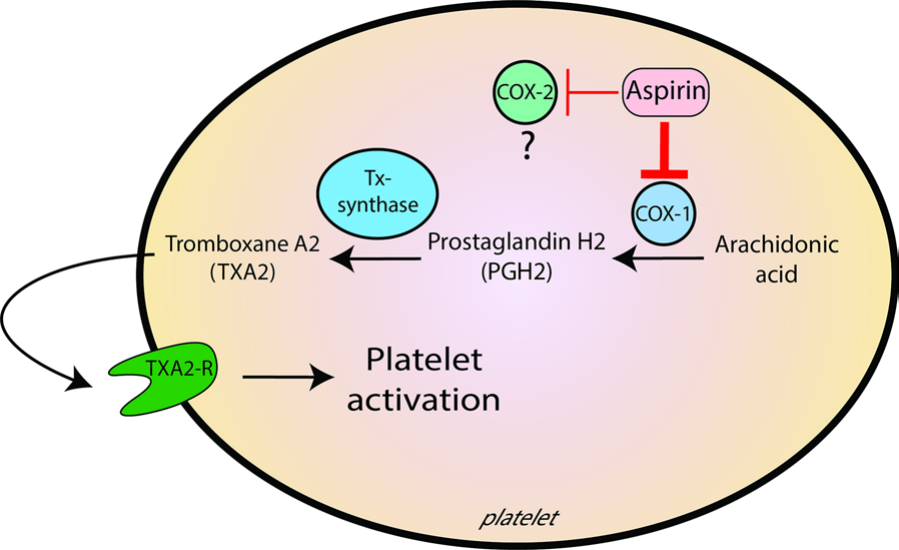
Aspirin’s target in platelets: aspirin inhibits cyclo-Oxygenase-1 (COX-1), limiting thromboxane A2 (TXA2) formation. The *thickness of line* represents the power of aspirin in inhibiting COX, which is about 200-fold higher for COX-1 than COX-2, whose role in platelet function is not clear

*The first*-*line drug* of the anti-aggregating therapy is *aspirin*, which is used in primary and secondary prevention of cardiovascular accidents. As extensively reviewed [6, 7], aspirin inhibition of platelet function is mediated by the permanent inactivation -due to acetylation of some serine domains- of Cyclo-Oxygenase-1 (COX-1), an enzyme constitutively expressed in platelets, which converts arachidonic acid into Prostaglandin H2 (PGH2), the substrate of the Thromboxane (TX)-synthase, which forms TXA2, a very powerful aggregating agent (Fig. 1). Aspirin is about 200-fold more powerful in inhibiting COX-1 than COX-2, which is inducible by inflammatory stimuli and is present in newly formed platelets. Indeed, in platelets the inducible COX-2 normally seems not expressed, though small quantities are detectable in mature platelets [8]. Therefore, the role of COX-2 in platelet function is not clear and it seems that the selective inhibitors of COX-2 only slightly reduce platelet TXA2 formation [9]. Indeed, it has been reported that the very low COX-2 expression in platelets does not influence the effect of aspirin [10].

Inhibition of COX-1 in platelet requires low doses of aspirin and platelet aggregability is irreversible as it lasts for the lifetime of platelets, which is of about 10 days. Aspirin benefits exceed the inhibition of TXA2, since it may increase platelet nitric oxide (NO) synthesis, protects NO from its inactivation, improves endothelial dysfunction, exerts anti-inflammatory effects [11].

## Oral anti-aggregant agents function on platelets

**Fig. 2:**
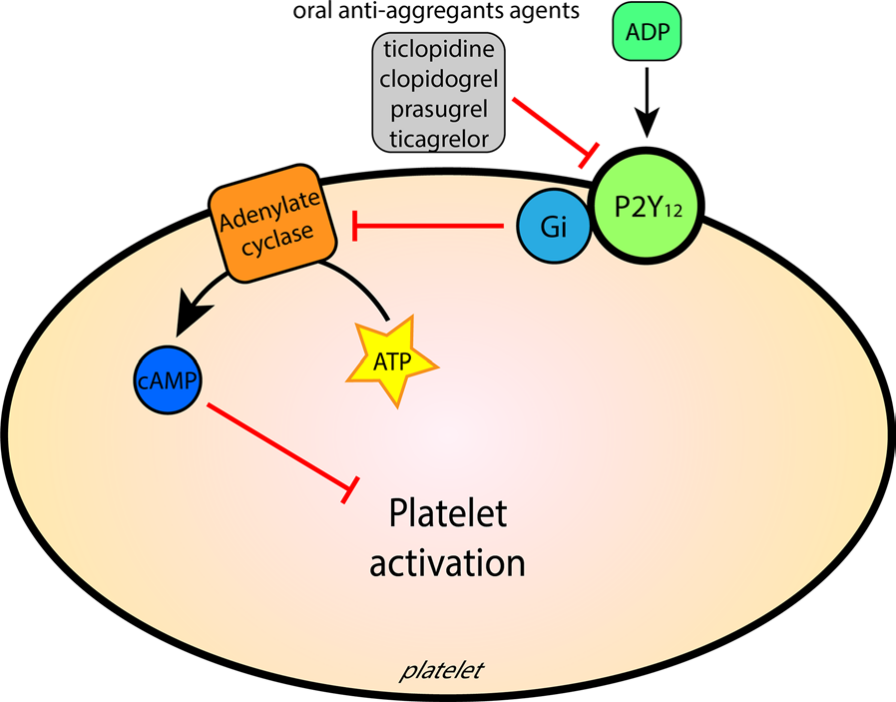
Target of oral anti-aggregant drugs in platelets: thienopyridine (clopidogrel and prasugrel) and non-thienopyridine (ticagrelor and cangrelor) directly or indirectly inhibit ADP receptor P2Y_12_, thus limiting adenylate cyclase inhibition and platelet aggregation

Either for secondary prevention or for subject undergoing a revascularization procedure, *oral anti*-*aggregant agents* have been proposed in the last years: ticlopidine, clopidogrel, prasugrel and ticagrelor. All these agents (either classified as thienopyridine or as non-thienopyridine) act directly or indirectly inhibiting adenosine diphosphate (ADP) receptor P2Y_12_ (Fig. 2). Although improved in the more recent compounds, the onset of anti-aggregating effect of these oral agents may be delayed because they are slowly adsorbed and may need to be converted to active substances by the liver. Thienopyridines (clopidogrel and prasugrel) require hepatic P450-mediated conversion of the pro-drug into its active metabolite [12]. Non-thienopyridine P2Y_12_ inhibitors (ticagrelor and cangrelor) are directly active, but have a long absorption time after oral administration, particularly in patients receiving opiate analgesia. This delayed onset of activity may be an issue in the clinical context in which it is necessary to minimize the door-to-balloon time. In order to render more effective percutaneous coronary intervention (PCI) an *intravenous* P2Y_12_ antagonist (*i.e.* cangrelor) may be considered. Cangrelor effects are very rapid and reach a very high anti-aggregation effect within a few minutes after *i.v.* administration. All these agents prevent the activation P2Y_12_, thus avoiding the activation of an inhibitory G protein (Gi) by ligands. Consequently, adenylate cyclase may increase the intra-platelet concentration of cyclic adenosine monophosphate (cAMP). Indeed, cAMP levels are inversely correlated with platelet activation conditions, and a cAMP decline contributes to activate platelet aggregation.

*Combining the COX inhibitor aspirin with a P2Y*_*12*_ *receptor inhibitor* is recommended in many clinical situations [13]. In particular, this dual antiplatelet association is recommended by the clinical guidelines in the current treatments of acute coronary syndromes (ACSs). Platelet inhibitors are usually given to the patients at the time of diagnosis just prior admission to the cardiac catheter laboratory and may be prescribed for long time treatments in the follow-up. The primary physiological and pharmacological focus of these drugs has been directed towards their ability to affect the blood rheology. These drugs effectively blunt platelet aggregability and reduce the risk of stent thrombosis. However, it has been suggested that when combined with a high level of P2Y_12_ blockade the net effect of higher doses of aspirin could be removal of anti-thrombotic and vasodilating prostanoids and so a lessening of the anti-thrombotic effectiveness of the treatment [14]. Therefore, this association must be used with cautions. It is suggested to include in the management of ACS the definition of platelet function, in addition to classical risk factors, in order to set a personalized therapy [13].

## Aspirin resistance in general population

Even if aspirin is a relevant tool to prevent cardiovascular events, as shown by meta-analysis of randomized trials [15, 16], the occurrence of “aspirin resistance” has been described, both as aspirin incapacity to protect individual patients from cardiovascular events (*i.e.,* clinical aspirin-resistance) [17–19] and as aspirin failure to induce a given degree of inhibition of platelet responses to agonists or TXA2 synthesis (i.e., laboratory-based aspirin-resistance*)* [20]. Indeed, “laboratory aspirin-resistance” is correlated to the risk of myocardial infarction and cerebrovascular events, leading to death [21–23].

Surprisingly, the role of the poor responsiveness to aspirin in affecting cardiac ischemia/reperfusion (I/R) setting has not been deeply investigated so far.

## P2Y_12_ inhibitor resistance in general population

Clinical efficacy may be reduced not only for aspirin, but also for P2Y_12_ inhibitors. The high inter-individual variability to *clopidogrel* inhibitory effects represents a clinical limitation: about 1 on 4 patients shows a suboptimal response to the drug [24]. Genetic and environmental factors can influence the absorption and/or the conversion of clopidogrel to its active metabolite. The most important cause of clopidogrel resistance has been attributed to the inability of cytochromes to metabolize this pro-drug to its active metabolite in the liver. However, it has been postulated by some authors that an imbalance between pro-inflammatory and anti-inflammatory cytokines due to altered cytochrome P450 genotypes may contribute to impair platelet aggregability, rather than alter the formation of the active metabolite of clopidogrel [25].

This drug resistance may be overcome with either high doses of elinogrel, cilostazol, clopidogrel, prasugrel, ticagrelor or Gp IIb/IIIa inhibitors [26]. The problems pertaining the P2Y_12_ inhibitors resistance are mainly described for clopidogrel. For instance, switching from clopidogrel to prasugrel was superior to standard treatment with clopidogrel for the achievement of optimal antiaggregation within the first 24 h post-angioplasty [27].

For details on other drugs the reader is kindly redirected to specific recent literature for drug resistance issues [26, 28].

## Antiplatelet therapy in type 2 diabetes

### The aspirin resistance in diabetes

The policy of antiplatelet therapy in T2DM is still a matter of intense debate. Patients with CVDs or with high cardiovascular risk profile, because of one or more comorbidities, such as diabetes, hypertension and/or hyperlipidemia, need early detection and appropriate therapies.

*In primary prevention* of cardiovascular events in T2DM potential benefits of aspirin are controversial. An Expert Consensus document has established that in primary prevention aspirin administration is not associated with a significant reduction in risk of non-fatal and fatal acute myocardial infarction (AMI) and, for this reason, is not recommendable in patients with T2DM at low cardiovascular risk [29].

As said aspirin resistance is not uncommon in the general population, and it has been described in great prevalence in obese and insulin-resistant patients [30, 31]. Of note, in the diabetic population aspirin resistance is particularly prevalent, inducing doubt about usefulness of aspirin for the cardiovascular prevention in this condition [32–34]. In fact, placebo-controlled studies and post hoc analyses consistently showed that diabetic patients receive less benefit from aspirin therapy than the non-diabetic population [35, 36]. In particular, a post hoc analysis revealed that in diabetic patients aspirin treatment did not cause a significant reduction in the risk of CVDs as opposed to subjects with other cardiovascular risk factors [37]. This analysis considered a subgroup of diabetic patients enrolled in the Italian Primary Prevention Project: a project evaluating the effects of low-dose aspirin, 100 mg/day, in the prevention of cardiovascular endpoints in patients with one or more risk factors [38]. Recently, a Japanese randomized trial (JPAD) has confirmed that aspirin fails to reduce the risk of cardiovascular events in primary prevention, but increases the risk of gastrointestinal bleeding [39].

*In secondary prevention* and in high-risk patients with previous AMI, *aspirin* may reduce the risk of future events by about 20% [40], thus justifying therapeutic guidelines recommendation on aspirin treatment for prevention of cardiovascular events in adults with diabetes and established CVDs. Of note, elevated fasting plasma glucose is associated with increased leukocyte-platelet aggregate rates, but high risk T2DM patients, without prior ischemic events, may have normal blood platelet functionality profiles [41]. These observations can explain why aspirin has no benefit in primary prevention of well-controlled diabetes, but is in some way only effective in secondary prevention (i.e. after ischemic events).

Indeed, *diabetes* is associated with reduced rates of responsiveness to aspirin and this contributes to explain why the reduction of the cardiovascular event risk in diabetic patients is lower than in non-diabetic subjects [17]. The high prevalence of poor responders to aspirin among diabetic individuals (1 in 4 have high on-treatment platelet reactivity while using aspirin daily) could therefore play a role in the poor cardiovascular outcome in diabetes [42–44]. Furthermore, it is possible that a great number of diabetic subjects are exposed to the gastrointestinal side effects of aspirin without experiencing the protective cardiovascular effects of the drug.

The mechanisms involved in platelet resistance to aspirin in diabetic patients are not completely clarified, even if different mechanisms have been hypothesized [45].

### P2Y_12_ inhibitor resistance in diabetes

As said, the concept of “resistance” has been described also for the P2Y_12_ inhibitor clopidogrel, and it has been associated with both modifiable and non-modifiable risk factors, including T2DM, body mass index, age, gender, smoking, and genetic polymorphisms, such as insulin receptor substrate-1 polymorphisms [46].

Particularly, in T2DM, studies have shown that a defect in the mechanisms through which insulin interferes with signalling by the P2Y_12_ receptor could contribute to platelet hyper-reactivity [47]. However, a stronger association between poor clinical outcomes of patients on treatment with clopidogrel and low platelet inhibition was found in T2DM patients compared with non-T2DM patients: [48] there are still conflicting reports linking low drug response and adverse interactions with clinical outcomes. A recent meta-analysis of published trial data did not suggest a different efficacy of clopidogrel in people with *vs* without diabetes [28].

Nevertheless, these findings do not preclude that both diabetic and non-diabetic subjects could have less-than-expected clinical benefits from clopidogrel treatment. For example, using a loading dose of clopidogrel, rather than small daily doses, was not enough for overcoming enhanced platelet reactivity in subjects with T2DM, suggesting that more effective anti-platelet drugs are necessary for such patients [49]. Of note, when these drugs, as well as aspirin, fail to protect individual patients from cardiovascular events, frequently costly surgical operations (e.g. coronary artery bypass, balloon angioplasty and stenting) are required to treat CVDs. Therefore, further studies are necessary to better understand the potentially different benefit-to-risk profile of subjects treated with anti-aggregant therapies.

## Influence of TD2M on cardioprotection and on IRI

CVDs are the number one cause of death: more people die from CVDs than from any other cause. CVDs account for about 30–35% of all global deaths. Of these 18 million deaths, about 45% were due to coronary heart disease (CHD) and 35% were due to stroke. About 75% of cardiovascular deaths occur in low and middle income countries. Of the 16 million deaths, below the age of 70, due to non-communicable diseases, 82% are in low and middle income countries and 37% are caused by CVDs [50]. The immigration from these countries is increasing and cost/effective therapies are much needed.

In patients with ST elevation myocardial infarction (STEMI), prompt reperfusion with primary PCI is the most recommendable intervention to limit infarct size. However, it is now clear that *IRI are due to both ischemia and reperfusion*. Indeed, *reperfusion injury* (the cell death resulting from the recovery of oxygen and blood supply) can contribute to about 50% of the final myocardial infarct size, both in experimental and clinical studies [51–53]. Limitation of IRI represents a currently unmet clinical need.

Indeed, significant myocardial necrosis still occurs despite timely reperfusion, and its extent is the main prognostic determinant following PCI. Different approaches to cardioprotection have been proposed to limit infarct size in patients with STEMI during primary PCI. Among these, intravenous or intracoronary injection of drugs or cells have been found safe and effective in experimental and clinical studies. However, further studies are needed to obtain satisfactory clinical results.

### Ischemic conditioning

In order to introduce the important concept of cardioprotective pathways and the concept of reperfusion injury, we report some brief notes on the cardioprotective mechanisms induced by ischemic conditioning. These brief notes are useful to better understand the following paragraphs. The reader is kindly redirected to specific literature for more details regarding the cardioprotective pathways activated by conditioning procedures [52–55].

*Ischemic pre*- *and postconditioning* are potent cardioprotective procedures to reduce infarct size due to myocardial I/R. It seems that these two conditioning protocols target reperfusion injury [52]. These two protocols trigger complex signalling pathways which start from the release of ligands released by ischemic and post-ischemic myocardium. This ligands-receptors interaction activates complex cascades including membrane G-protein, growth factor receptors, signalling enzymes, such as nitric oxide synthase (NOS), protein kinase C and G (PKC and PKG), as well as ATP-sensitive potassium (KATP) channels, reactive oxygen species (ROS), TNF-α and sphingosine-1-phosphate (S1P) [56]. The final effector is likely the mitochondrial permeability transition pore (mPTP) where the signalling cascade prevents pore formation leading to protection [57]. This signalling pathway could represent a therapeutic target to protect patients with AMI.

However, to date the conditioning interventions showing efficacy in reducing IRI in laboratory have been unsuccessful in clinical context. The reasons for the inability to translate positive results obtained in the experimental laboratory to the clinical scenario have been discussed in several recent reviews: among the plethora of reasons, the presence of comorbidities and related therapies in humans, not considered in animal experiments, have been considered several times [51–53, 58–60].

### The increased susceptibility to IRI in diabetes

Diabetes represents an important comorbidity in the patients presenting with an ACS. The majority of both pre-clinical and clinical data demonstrates that the diabetic heart is among the most susceptible to IRI and that the cardioprotective effects of ischemic and pharmacological conditioning are compromised in the presence of diabetes. Therefore, diabetic subjects have greater probability to develop an acute event and perhaps a limited possibility to reduce infarct size by cardioprotective interventions [61]. The increased susceptibility to IRI in the setting of diabetes mellitus is due to several mechanisms, including alteration at mitochondrial level, altered production of ROS and impairment of antioxidant capacities at various intracellular and extracellular sites. The compromised protection is often associated with the deficient activation of prosurvival signalling pathways, such as Akt, ERK1/2, GSK3β, Janus kinase 2 and STAT3. Interestingly, the persistence of hyperglycaemia in diabetic conditions has been shown to decrease generation and release of NO and to increase the expression and activity of “phosphatase and tensin homolog deleted on chromosome 10″ (PTEN), which may contribute to inhibit Akt/NO pathway [62–66].

Also, a reduced availability of adenosine may contribute to the loss of cardioprotective efficacy by conditioning protocols in diabetes [67]. Finally, sarcolemmal and mitochondrial KATP channel dysfunction, as well as prolonged mPTP opening and ROS-induced ROS release in response to Ca^2+^ overload in the diabetic myocardium have been described [68–70].

Therefore, it is likely that the mitochondria function and redox state have fundamental roles in understanding the susceptibility to CVDs of patients with diabetes. An in-depth description of these aspects of diabetes sensitivity to IRI and loss of mechanisms of protection can be found in several recent reviews [60, 67, 71].

Here we will consider the putative role of anti-platelet therapies in interfering with cardioprotective interventions in experimental conditions considering the intricate role of diabetes and platelets in determining IRI.

## Influence of anti-platelet therapies on IRI

Blockade of platelet aggregation during primary PCI for AMI is a standard care to inhibit intravascular coagulation and to minimize stent re-thrombosis. Indeed, anticoagulant therapy during primary PCI for AMI is routinely applied, and a description of this approach is beyond the aim of the present mini-review, the reader is kindly redirected to specific literature for issues regarding the anticoagulant effects of these drugs [72, 73].

Here we consider the ability of P2Y_12_ inhibitors to ameliorate myocardial response to ischemia/reperfusion challenging, which are part of the so-called *pleiotropic properties of anti*-*platelet therapies* investigated by Cohen and Downey group. It is likely that nowadays all patients with ACSs are treated with platelet P2Y_12_ receptor antagonists. It has been proposed that those patients receiving P2Y_12_ receptor antagonists are already cardioprotected through the conditioning pathways outlined above. Whether this cardioprotective effect is due to an amelioration of platelet function or is due to a direct effect on myocardium, is under investigation. Whatever the case, if it is true that patients receiving P2Y_12_ receptor antagonists are indeed already benefiting from conditioning cardioprotection, to further decrease infarction interventions which protect by different mechanisms are necessary. Indeed, it has been reported that clopidogrel and cangrelor protect the rabbit heart against infarction [74]. For both drugs the mechanism is not due to inhibition of intravascular coagulation, but involves signalling pathways activated during reperfusion to prevent reperfusion injury. Therefore, it is likely that patients receiving P2Y_12_ inhibitors before PCI may already be conditioned, thus explaining failure of recent clinical trials evaluating postconditioning procedures [56, 75].

If antiaggregating therapy protects via a similar signalling pathway of conditioning interventions, then it would make any other conditioning procedure that protects via the same signalling pathway redundant. Indeed, it has been proved that ischemic pre- or post-conditioning do not add any further protection to the heart of experimental animals protected with *cangrelor.* Nevertheless, in these animals treated with cangrelor, procedures and drugs that use different mechanisms such as mild hypothermia or cariporide, a Na^(+)^-H^(+)^ exchange blocker, display additional cardioprotective effects. The important consequence of these results is that patients with AMI who are treated with anti P2Y_12_ agents may already be in a sort of “conditioned state”. Thus, explaining why conditioning procedures had only a modest if any effect in recent clinical trials. These studies clearly indicate that future research should identify interventions and procedures that can enhance the protection obtained by antiaggregating therapy and should find a way to optimize P2Y_12_ inhibition at reperfusion in all patients [74, 76].

Recently it has been reported that increasing DNA glycosylase/AP lyase repair enzyme activity confers cytoprotection in several injury models [77]. Therefore, the effects of EndoIII, a fusion-protein construct that conveys Endonuclease III, a DNA glycosylase/AP lyase, to the mitochondria, were studied in terms of infarct size reduction in myocardial IRI model. In this study, an *i.v.* bolus of EndoIII, just prior to reperfusion, reduced infarct size. The EndoIII-induced infarct limitation was comparable to that seen with P2Y_12_ receptor blocker, cangrelor. Importantly, the combination of EndoIII with cangrelor reduced infarct size in an extent that was greater than that observed with either cangrelor or EndoIII alone. Cardioprotection obtained with cangrelor but not that obtained with EndoIII was abolished by pharmacologic inhibition of adenosine receptors or phosphatidylinositol-3 kinase, suggesting different cellular mechanisms for these two interventions. It seems that EndoIII protects the heart from necrosis by avoiding the release of pro-inflammatory fragments of mitochondrial DNA (mtDNA) into the myocardium. Therefore, EndoIII and DNase have been proposed as agents that can be administered at reperfusion to add their protective effect to those of a P2Y_12_ blocker already used in patients, and thus should be theoretically effective in patients presenting with AMI. Of course, this hypothesis needs to be verified in appropriate clinical studies [77].

Studies in rodents and primate models [56, 74, 76] confirm that platelet P2Y_12_ receptor antagonists are cardioprotective, and that the mechanism is not due to inhibition of platelet aggregation, but to the ability of these agents to trigger the same signal transduction pathway of conditioning cardioprotection. The cardioprotective effects exerted by these drugs are a class-effect. In fact, either thienopyridine or non- thienopyridine P2Y_12_ inhibitors have shown similar cardioprotective effect when an adequate concentration of the agent was present in the blood at the moment of reperfusion [78, 79].

From above one can infer that antiplatelet therapy directly protects the myocardium against IRI. However, the subtitle mechanism(s) by which platelet P2Y_12_ inhibitors induce protection remains unclear. The Cohen & Downey group provided evidence that this cardioprotection is possible only if whole blood is present in the experimental preparation: while both ischemic postconditioning and cangrelor protect blood-perfused hearts, the drug does not protect buffer-perfused hearts, suggesting that the cangrelor requires blood constituents or factors to protect. These authors also used an anti-platelet antibody to make rats thrombocytopenic and to test if that blood-factor resides within the platelets. IRI were not different in thrombocytopenic rats, suggesting that preventing aggregation alone is not protective. While ischemic pre-conditioning could reduce infarct size in thrombocytopenic rats, the P2Y_12_ inhibitor cangrelor could not, strongly suggesting that it protects by interacting with some factor in the platelets. These authors confirm that platelet P2Y_12_ receptor antagonists protect the animal heart, not because of inhibited thrombosis, but through the activation of protective signalling. Ischemic preconditioning triggers the phosphorylation of sphingosine leading to the formation of S1P, which is protective. Therefore, these authors demonstrated that in rats treated with dimethylsphingosine to block sphingosine kinase, cangrelor was no longer protective. Thus, protective mechanisms activated by cangrelor also involve sphingosine kinase, thus demonstrating another analogy to conditioning protective mechanism [78, 79].

Importantly, differently from cardioprotection induced by pharmacological and ischemic conditioning, P2Y_12_ inhibitor-induced mechanisms can result in significant attenuation of IRI in animals with diabetes [79]. These last results open an interesting scenario on the role of platelets in cardioprotection: one may wonder whether there are differences on the effects of platelets on IRI depending on whether they are healthy or diabetic.

## Influence of healthy and diabetic platelets on IRI

There are clear evidences that healthy platelets have cardioprotective properties [80, 81]. Recently we have shown that the beneficial potentials of healthy platelets are lost by platelets derived from poorly controlled T2DM patients [82].

Platelet hyper-reactivity is a central cause of accelerated atherosclerosis and increased risk of cardiovascular events in diabetic patients. The mechanisms leading to altered platelet reactivity during diabetes are complex and not fully elucidated. Platelet dysfunction may be due to the fact that diabetes is associated with oxidative stress and inflammation. These conditions may also induce endothelial dysfunction which may promote activation of platelets by decreasing production of NO, thus attenuating platelet reactivity. Oxidative stress within platelets may exacerbate this effect by attenuating production and activity of NO and promoting platelet activation [83, 84].

Oxidative stress is considered a major determinant, not only of vascular complications, but also of suboptimal response to antiplatelet agents in T2DM [85]. Moreover, platelet activation and inflammation are reciprocally related: inflammation favours platelet activation that, in turn, induces inflammation [84, 86].

Furthermore, diabetic platelets have enhanced levels of platelet identifiers (CD41-PE, CD42b-PE) as well as markers indicating platelet activation (CD62P-PE and CD63-PE) [87]. Finally, ultrastructural and rheological modifications, as well as alteration in plasma components, such as reduction of CTRP9 concentration, have been described as crucial in platelets hyper-reactivity of diabetic subjects or animals [88, 89].

Overall, there are a number of alterations in diabetic platelets, but which ones play a key role in determining the impact of diabetes on platelet function, platelet-mediated effects and in platelet response to anti-aggregating therapy is still unclear.

Platelets are known to release a variety of factors on activation. These factors may contribute to the positive effects exerted by platelets in a variety of clinical and experimental conditions. For instance, platelets have been used for soft-tissue healing of low extremity ulcer [90, 91]. They have also been used in association with mesenchymal stem cells to promote bone regeneration [92–95]. In particular, perfusion of hearts during reperfusion with buffer containing washed rat platelets protected hearts against dysfunction from IRI [80]. However, several studies have suggested that platelets may contribute to initiation and propagation of myocardial IRI [96–98].

Nevertheless, in most of these studies in which platelets seem to play a pathological role there is a *concomitant pathological condition* (comorbidities) which may influence the outcome. In particular, it seems that a defective endothelial function may characterize many of these conditions. In fact, it has been proposed that in the exacerbation of myocardial IRI may play a pivotal role the interaction between platelets, blood cells and endothelial wall [99, 100].

**Fig. 3:**
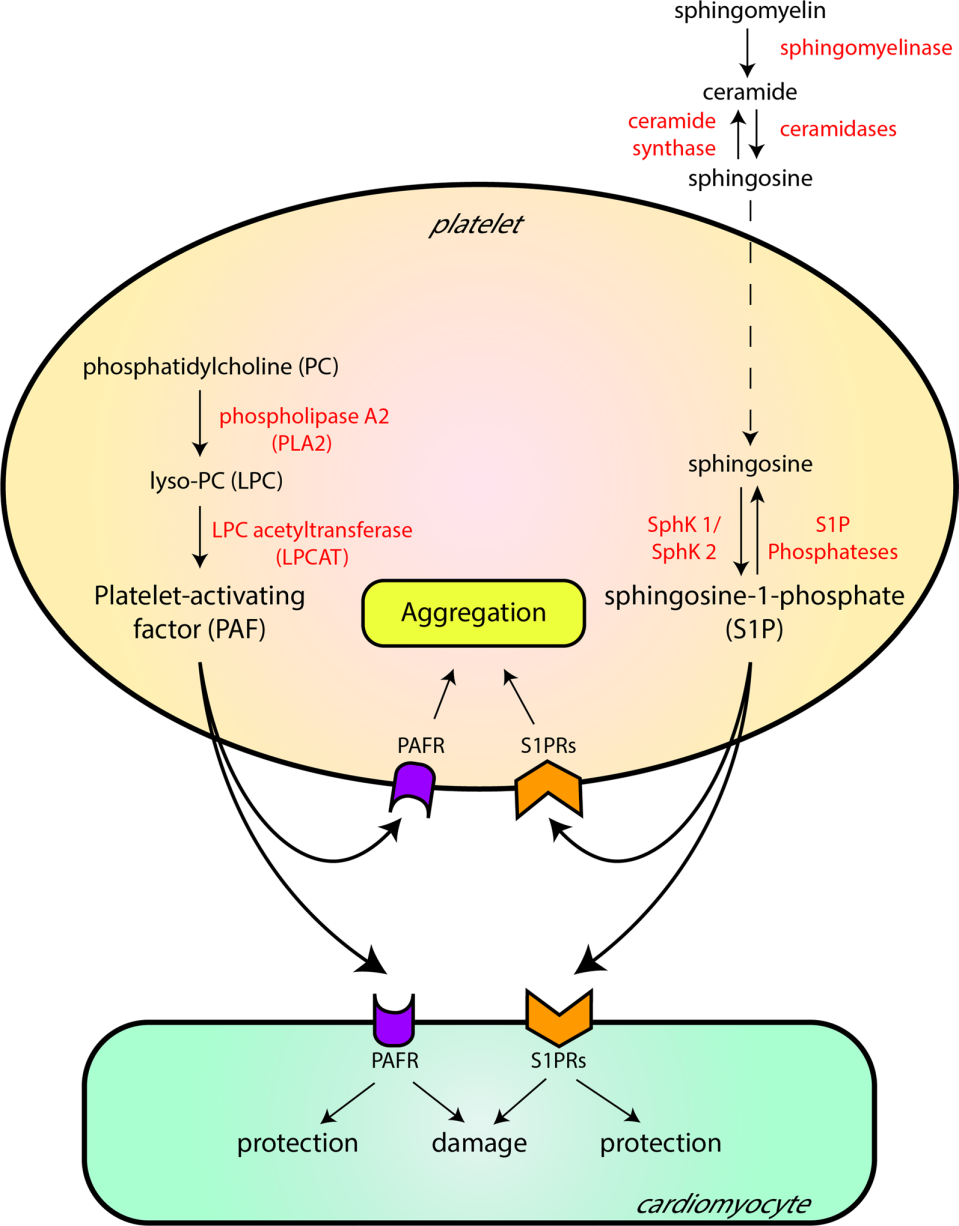
Among the factors released by platelets, some have double edged sword behavior. Depending on several factors (e.g. concentration and timing of infusion), they may induce both cardioprotection (i.e. preconditioning like effects) or injury to cardiomyocytes

Among the factors released by platelets, some have double edged sword behaviour. They may induce cardioprotection (i.e. preconditioning like effects) or may induce injury depending on several factors (e.g. concentration and timing of infusion) (Fig. 3). Among variable platelet factors that may display double-edged sword characteristics in the context of cardioprotection, there are Sphingosine-1-Phosphate (S1P), Platelet-activating factor (PAF) and Chemerin (CHE). We have shown that low concentrations of PAF activate the Reperfusion Injury Salvage Kinase (RISK) pathway, including PKC, Akt and NOS [101]. This pathway converges on mitochondria preventing mPTPs opening at reperfusion [102]. Moreover, we have preliminary evidences of S1P alterations in diabetic platelets, and CHE is a predictive marker of CVDs [103]. Indeed, CHE was described as a chemo-attractant for leukocyte expressing the chemokine-like receptor1 (CMKLR1 or ChemR23). However, recently, CHE has been identified as an adipokine associated with obesity, insulin resistance and metabolic syndrome [104]. Several cell types, including adipocytes and vascular smooth muscle cells produce CHE as a precursor converted into a bioactive form by a number of serine proteases. Platelets also are an important source of CHE, which becomes released in some pathologic conditions [105]. Elevated serum CHE levels are associated with ischemic heart disease and CVD in T2DM patients [106, 107]. Intriguingly, plasma CHE levels are reduced in CVD patients treated with aspirin [108]. Thus, given its dual role, CHE deserves to be studied as a possible metabo-inflammatory player in obesity-related disorders, such as T2DM and CVD.

As said, preliminary experiments have demonstrated beneficial role of healthy platelets in the context of myocardial I/R. These beneficial potentials of healthy platelets are lost in platelets derived from T2DM patients. We have shown that the effects of platelet-pre-treatment on IRI depend on their reactivity status as represented by the trend to aggregate which correlates with redox status of platelets [109].

We do not know whether or not the platelets of T2DM patients taking aspirin or P2Y_12_ inhibitor regain their cardioprotective properties and/or if this occurs only when platelets do not display anti-aggregating resistance. We are working on this hypothesis and hope to have an answer to this issue in the future.

## Conclusions and future perspectives

In summary: (1) diabetes confers a deleterious phenotype in patients presenting with ACSs; (2) diabetes may also be associated with altered platelet function and response to aspirin and other anti-platelet therapies, especially in the presence of comorbidities; (3) some P2Y_12_ inhibitors may remain protective in diabetic patients and animal models of diabetes. These are important observations not fully understood that require further studies.

Since the prevalence of diabetes in the general population is dramatically increasing and since diabetes negatively influences IRI and cardioprotective procedures, the management of ischemic heart disease as well as the continuation of the recent trend of improving cardiovascular outcomes from CHD will become increasingly difficult. To help to individuate additional drug targets to improve outcomes of CVDs further, particularly in those patients with comorbidities, such as diabetes, it is necessary a better comprehension of the differences and similarities between the protective mechanisms of the different cardioprotective strategies, in the various subpopulations of patients. In particular, comparative studies on the mechanisms of the ischemic conditionings, and on the emerging platelet-mediated cardioprotection induced by P2Y_12_ inhibitors are warranted. The intricate interaction between platelet function, antiplatelet drugs, coronary diseases and IRI in diabetes needs to be clarified for enhancing patients benefit from antiplatelet therapy and revascularization.

Future perspectives point toward an important role for miRNA in platelet function and therapy [110]. Indeed, recent studies revealed important roles for miRNA in platelet development, function and CVD [111]. miRNAs are present in platelet where they interfere with target such as c-AMP dependent PKA and receptors, including P2Y_12_ and glycoprotein IIb/IIIa. Moreover, activated platelets can release functional miRNA that can regulate gene expression in cells of the cardiovascular system [112]. Several platelet miRNA related to CVD, such as miRNA-21 and miRNA-126, are significantly modulated in T2DM and studies are in progress to identify a miRNA-based signature associated with T2DM and its CVD complications [113]. Interestingly, platelet miRNA are responsive to antiplatelet therapy and may represent valid biomarker for the response to therapy as emerged in recent studies showing that aspirin resistance can potentially be identified by miR-92a and miR-19b-1-5p levels in plasma [114, 115]. Based on these observations, miRNA may become a valuable tool to provide diagnostic and prognostic information about T2DM, coronary artery disease complications and therapy responsiveness.
